# Engaging family supporters of adult patients with diabetes to improve clinical and patient-centered outcomes: study protocol for a randomized controlled trial

**DOI:** 10.1186/s13063-018-2785-2

**Published:** 2018-07-24

**Authors:** Ann-Marie Rosland, John D. Piette, Ranak Trivedi, Eve A. Kerr, Shelley Stoll, Adam Tremblay, Michele Heisler

**Affiliations:** 1 VA Pittsburgh Center for Health Equity Research and Promotion, University Drive (151C), Building 30, 2nd Suite 2A128, Pittsburgh, PA 15240-1001 USA; 2 Department of Internal Medicine, University of Pittsburgh, 230 McKee Place, Pittsburgh, PA 15213 USA; 3 Veterans Affairs Center for Clinical Management Research, VA Ann Arbor Center for Clinical Management Research, 2215 Fuller Road, Ann Arbor, MI 48105 USA; 4 Department of Health Behavior and Health Education, School of Public Health, University of Michigan, 1415 Washington Heights, 1700 SPH I, Ann Arbor, MI 48109 USA; 5 Center for Innovation to Implementation, VA Palo Alto Center for Innovation to Implementation, 795 Willow Road, 152MPD Building 324, Palo Alto, CA USA; 6 Department of Psychiatry and Behavioral Sciences, Standford University Medical School, 401 Quarry Road, Stanford, CA 94305-5717 USA; 7 Department of Internal Medicine, University of Michigan Medical School, 1600 Plymouth Road, Ann Arbor, MI 48109 USA; 8 Department of Ambulatory Care, VA Ann Arbor Healthcare System, 2215 Fuller Road, Ann Arbor, MI 48105 USA

**Keywords:** Patient-centered medical home (PCMH), Diabetes mellitus, Caregiver, Social support, Self-management (SM), Patient activation, Interactive voice response, Action planning, Health coaching, Automated calls

## Abstract

**Background:**

Most adults with diabetes who are at high risk for complications have family or friends who are involved in their medical and self-care (“family supporters”). These family supporters are an important resource who could be leveraged to improve patients’ engagement in their care and patient health outcomes. However, healthcare teams lack structured and feasible approaches to effectively engage family supporters in patient self-management support. This trial tests a strategy to strengthen the capacity of family supporters to help adults with high-risk diabetes engage in healthcare, successfully enact care plans, and lower risk of diabetes complications.

**Methods/design:**

We will conduct a randomized trial evaluating the CO-IMPACT (Caring Others Increasing EnageMent in Patient Aligned Care Teams) intervention. Two hunded forty adults with diabetes who are at high risk for diabetes complications due to poor glycemic control or high blood pressure will be randomized, along with a family supporter (living either with the patient or remotely), to CO-IMPACT or enhanced usual primary care for 12 months. CO-IMPACT provides patient-supporter dyads: it provides one coaching session addressing supporter techniques for helping patients with behavior change motivation, action planning, and proactive communication with healthcare providers; biweekly automated phone calls to prompt dyad action on new patient health concerns; phone calls to prompt preparation for patients’ primary care visits; and primary care visit summaries sent to both patient and supporter. Primary outcomes are changes in patient activation, as measured by the Patient Activation Measure-13, and change in 5-year cardiac event risk, as measured by the United Kingdom Prospective Diabetes Study cardiac risk score for people with diabetes. Secondary outcomes include patients’ diabetes self-management behaviors, diabetes distress, and glycemic and blood pressure control. Measures among supporters will include use of effective support techniques, burden, and distress about patient’s diabetes care.

**Discussion:**

If effective in improving patient activation and diabetes management, CO-IMPACT will provide healthcare teams with evidence-based tools and techniques to engage patients’ available family or friends in supporting patient self-management, even if they live remotely. The core skills addressed by CO-IMPACT can be used by patients and their supporters over time to respond to changing patient health needs and priorities.

**Trial registration:**

ClinicalTrials.gov, NCT02328326. Registered on 31 December 2014.

**Electronic supplementary material:**

The online version of this article (10.1186/s13063-018-2785-2) contains supplementary material, which is available to authorized users.

## Background

The prevalence of diabetes in the USA is growing [1, 2], and many adults with diabetes are at high risk for diabetes complications due to uncontrolled risk factors [3, 4]. Despite high-quality diabetes care delivery, 20–30% of patients with diabetes have poor glycemic control or poor blood pressure (BP) control [5]. Patients with uncontrolled risk factors are at high risk for disabling and costly diabetes complications, including stroke, heart attack, amputation, kidney failure, or blindness. To reduce diabetes complications, these “high-risk” patients are given treatment regimens that are complicated and often difficult to follow in day-to-day life. In addition, these patients must effectively communicate and coordinate with multiple medical providers and proficiently navigate the healthcare system. In light of these complex care needs, high-risk patients often need more support than the healthcare systems have the capacity to offer.

One relatively untapped resource for this support is a patient’s natural social network of family and friends. Three out of four adults with diabetes reach out to an unpaid family member or friend (a “family supporter”) for ongoing help with diabetes management [6, 7]. These supporters assist patients in engaging in activities directly related to successful diabetes management, including medication management and adherence, tracking home glucose and BP measurements, maintaining a healthful eating plan, and being physically active [6–8]. Family supporters also often help patients make key decisions about their diabetes management, such as how to address medication side effects [9]. Typically, 50–60% of family supporters are spouses, and most of the rest are family members who do not live with the patient (such as adult children) [6, 10, 11]. Prior research has shown that chronically ill patients with low health literacy, multiple comorbidities, and comorbid depression involve family supporters in their care more often [12–14].

**Fig. 1:**
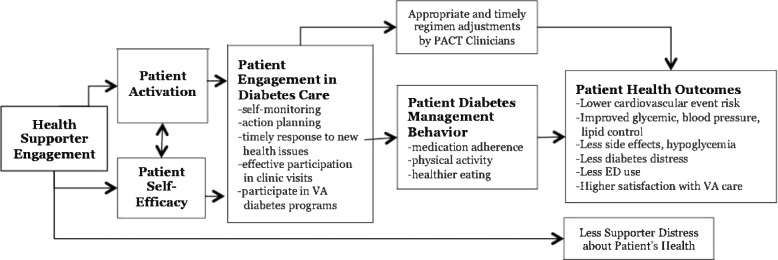
Theoretical model

Family and friend supporters are uniquely poised to provide personalized, frequent, and ongoing support for health management. Trusted family supporters often already interact frequently with patients in their home environment as part of established long-term relationships. Family and friends have unique vantage points to identify and understand patients’ struggles with health management and then intervene effectively. In fact, research studies have consistently shown that chronically ill patients with family supporters have better self-management and long-term health outcomes [15–18]. For patients with diabetes, higher levels of family support are linked with better glycemic control and lower mortality [15]. In other chronic conditions that require significant self-management, such as cardiac disease and heart failure, higher levels of social support are linked to lower rates of recurrent cardiac events and hospitalizations [16, 17]. There is strong evidence that social support acts on chronic disease outcomes largely through improved patient self-management behaviors (see the theoretical model in Fig. 1) [19].

Recent Institute of Medicine reports on aging and caregiving have concluded that family caregivers should be explicitly incorporated into healthcare delivery [20, 21]. Yet, healthcare systems lack formal mechanisms to involve family supporters in care. This is unfortunate, as these supporters could play a crucial role in helping patients effectively engage in behaviors to improve health. Increasingly common team-based models of healthcare, such as the patient-centered medical home (PCMH) — these team-based models are known as Patient Aligned Care Teams (PACT) — in the Veterans Health Administration (VHA) [22], provide opportunities to expand our concept of teamwork in healthcare to include not only allied healthcare professionals but also lay people such as family supporters. Prior studies indicate that family supporters are already highly involved in patients’ interactions with the healthcare system. About half of patients with diabetes are regularly accompanied by a supporter in the exam room for healthcare visits [12, 13], and 25% have had a supporter talk on the phone with their clinician in the last year [12]. Importantly, family supporters often help patients prepare questions before healthcare visits, assist patients in processing information and plans (“debriefing”) after visits, and help patients navigate health system services such as pharmacy fills and diabetes class enrollment [23].

Previous interventions aiming to leverage family support to improve disease management have generally engaged supporters in patients’ day-to-day health management through counseling or coaching [24, 25]. Such interventions have demonstrated improvements in dietary behavior among patients with heart failure [26] and physical activity among obese patients [27]. However, no published interventions or clinical programs we are aware of have focused on helping family supporters boost chronically ill patients’ engagement in clinical care and medical self-care (e.g., medication adherence).

One promising lever for helping family supporters be more effective at improving patient health is by training them to boost patient activation. “Activated” patients are those who have the “skills and confidence to become actively engaged in their health and healthcare” [28]. Activation includes the ability to share in decision-making with healthcare providers, monitor and self-manage symptoms, and access care in an appropriate and timely way. Highly activated patients have better health behaviors (including adherence to medications, regular self-monitoring at home, physical activity, and healthful eating) and health outcomes (including lower body mass index, hemoglobin A1c (HbA1c), BP, and cholesterol) [29]. Increases in patient activation over time are linked to improvements in similar health behaviors and outcomes [30]. There are several reasons to hypothesize that family supporters can help increase patient activation. There are very strong links between social support and improved patient self-efficacy for self-care [14, 31–34], a concept closely related to patient activation. Higher social support is linked to activated self-management behaviors, such as increased self-monitoring [35, 36]. When supporters accompany patients to medical visits, the patients exhibit more activated behavior, including increased participation in decision-making with providers [10, 12, 13]. In prior studies, patients participating with a family supporter in an interactive voice response (IVR) self-management intervention were more engaged in the intervention than those who participated alone [37, 38].

There are several other promising methods to increase the ability of family supporters to positively affect the health of patients with diabetes. In our national survey of 760 family supporters of patients with chronic disease [39], supporters reported feeling limited by a lack of patient-specific information, such as changes in medication regimens or test results, as well as a lack of health system-specific information, such as the roles of healthcare team members or available diabetes programs [9]. Supporters also face significant challenges when helping patients prepare for, and debrief after, clinical visits. For example, patients often do not bring written questions for the doctor, and many are not confident they are reporting accurate visit information back to their supporter [23]. Twenty-eight percent of supporters reported that their patient-partner regularly discusses being confused about healthcare provider instructions [39]. Prior studies also indicate that family supporters have been less effective at influencing patients’ medical self-management tasks (e.g., medication adherence or blood glucose monitoring) than healthful lifestyles (e.g., healthful eating) [19, 35, 40], and therefore family supporters may benefit from training focused on increasing their knowledge of and comfort with medications and monitors.

Finally, family supporter effectiveness could also be boosted through more structured and action-oriented between-visit discussions with patients. In our national family supporter survey, we found that supporters discuss health with their patient-partners almost every time they talk, but approximately 30% were unsure what questions to ask or what advice to give about diabetes [9]. Supporters can make the most of these discussions when they have patient-specific information and when they use evidence-based support techniques, such as positive and autonomy-supportive statements and collaborative action planning and coping [41].

We designed an intervention that incorporates these promising evidence-based and stakeholder-informed methods to increase family supporter effectiveness in helping patients increase patient activation and manage diabetes successfully. The intervention, called Caring Others Increasing EngageMent in PACT (CO-IMPACT), provides coaching, tools, and information to family supporters and patients in the primary care setting. The overarching goal of this intervention is to structure and facilitate family supporter involvement in healthcare so that patients can become more actively engaged in their care and improve their diabetes management and outcomes. CO-IMPACT approaches family supporters as part of the patient’s healthcare team, helping to support the patient in patient-led self-management. CO-IMPACT will address key limitations to a supporter’s potential to be effective in this role by providing supporters with the following: ongoing information about their patient-partner’s health status and treatment plan; ways to help patients identify and engage in appropriate healthcare system services; structured pre- and post-primary care visit information that can improve supporter-patient discussions about diabetes plans; and guidance to supporters on evidence-based communication techniques such as autonomy-supportive communication. The CO-IMPACT approach does not focus on diabetes management education, but instead focuses on underlying skills that family supporters can use over time to help patients improve activation in care and successful self-management of health conditions.

We have designed CO-IMPACT so that it can be incorporated into care delivered by PCMH team members, such as nurse care managers or health coaches, and to take advantage of technology available to healthcare teams, such as patient portals and mobile-health automated monitoring systems. Our central hypothesis is that providing healthcare engagement tools to both health supporters and patients will increase patient activation and improve management of diabetes complication risks. We will evaluate the impact of CO-IMPACT on patient activation, patient health behaviors, and physiologic changes in diabetes complication risk factors, while at the same time measuring the intervention’s impact on family supporters.

## Methods/design

### Overall design and aims

**Fig. 2:**
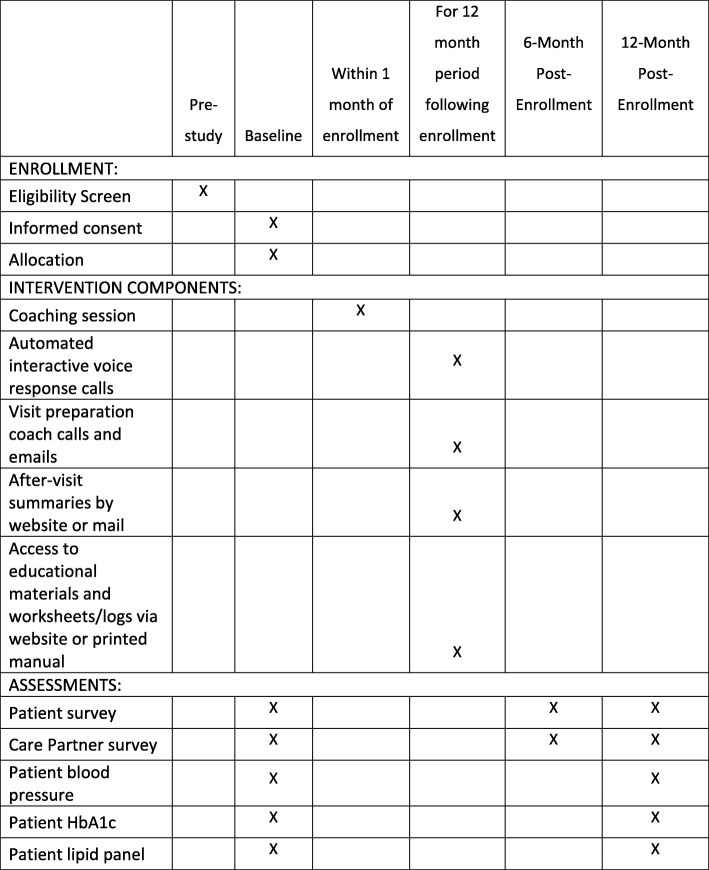
Schedule of standard protocol items

**Fig. 3:**
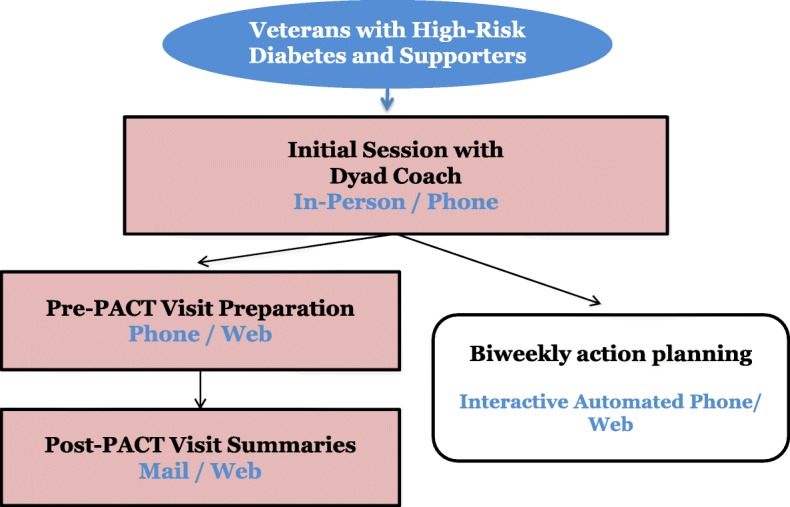
Flow of intervention components

This will be a randomized controlled trial evaluating the superiority of the CO-IMPACT intervention over enhanced usual care. Two hundred forty patients with diabetes receiving primary care at the VHA who are at high risk for diabetes complications and who have a family supporter involved in their care (called the patient’s “Care Partner”) will be recruited along with the family supporter. Patients will be identified for recruitment from a data warehouse containing VHA patient health record data, and patient-supporter dyads will be randomized to CO-IMPACT or usual PACT primary care. Outcomes will be measured at baseline, 6 months, and 12 months post-enrollment via patient and supporter surveys, and patient laboratory tests, vital signs, and medical records. Figure 2 indicates the schedule of standard protocol items, and Fig. 3 outlines the flow of intervention components.

The study’s specific aims are to:

1. Determine the effect of the CO-IMPACT intervention on patient engagement in treatment and on health behaviors among patients at high risk for diabetes complications. We hypothesize that CO-IMPACT will significantly increase patient activation, as measured by the Patient Activation Measure-13 (PAM-13) compared to usual VA PACT care.
2. Determine the effect of the CO-IMPACT intervention on health risks among patients at high risk for diabetes complications. We hypothesize that CO-IMPACT will significantly decrease patients’ 5-year cardiovascular event risk, as measured by the United Kingdom Prospective Diabetes Study (UKPDS) cardiac risk score (which includes HbA1c, non-fasting lipid levels, and BP) [42] compared to usual PACT care
3. Evaluate how the characteristics of patients, family supporters, and their relationships mediate and moderate the effects of CO-IMPACT. We hypothesize that higher levels of family supporter participation in CO-IMPACT intervention components will lead to greater improvements in patient activation and patient cardiac risk.

#### Setting

Participants will be recruited from among patients receiving care at two Veterans Affairs (VA) primary care clinics. One is a large, multiprovider clinic linked with a tertiary healthcare center; the other is a large, free-standing, community-based outpatient clinic. The study contacts will take place mainly via the Internet, telephone, and mail, enhanced by an initial coaching session in the outpatient clinic. The protocol is designed at each stage to accommodate family supporters who live with the patient, apart from but close to the patient, or at a distance from the patient. These accommodations for family supporter participants include phone-based recruitment, screening, and consent processes, and Internet- and phone-based intervention participation options [43].

#### Participants and recruitment

We will identify potentially eligible patients via the VHA’s Corporate Data Warehouse (CDW), a national repository of data extracted from VA electronic medical record systems. Patients must be receiving care at one of the recruitment sites and be between 30 and 70 years old, as the study focuses on (1) family interactions among mature adults rather than teens or those who have had type 1 diabetes since their youth, and (2) goals for reducing 5-year diabetes complication risk that might be altered for more elderly patients with more limited life expectancy or who are at higher risk from tighter diabetes control. Eligible patients will have (1) a diagnosis of diabetes based on diagnoses from one inpatient or two outpatient encounters, or a diabetes medication prescription (at least one > 3-month prescription for insulin or an oral diabetes medication other than metformin), (2) a usual VA primary care provider (who is not a medical resident/trainee nor a geriatric specialist) and at least two visits to their usual VA primary care clinic in the previous 12 months, (3) poor glycemic control (last HbA1c within 9 months > 8%) or poor BP control. Poor BP control is defined as (1) more than one BP reading in last 9 months: most recent systolic blood pressure (SBP > =150 mmHg, and mean SBP over 9 months > = 150 mmHg; or (2) if only one BP reading in the last 9 months: last SBP in last 6 months > = 160 mmHg; or (3) last diastolic BP or mean diastolic over 9 months not <= 65 mmHg. If multiple BPs were recorded in 1 day, the lowest one will be used. BPs recorded on days with encounters in the emergency department, urgent care, or surgical clinics; medical procedures; or inpatient days will not be used to determine patient eligibility. Patients will be excluded from the trial if they have a serious mental illness (bipolar, schizophrenia, delusional disorders, or other psychoses), dementia, moderate to profound intellectual disability, or active substance abuse as determined by encounter codes in a single inpatient or outpatient encounter in the last 2 years.

Potentially eligible patients who meet the preceding initial criteria will be sent an introductory letter informing them about the study and inviting them to learn more about participating. The letter will give the participant a method for opting out of further contact. In the absence of such notification, 7–10 days after the letter is expected to arrive, study staff will call patients to explain the study in more detail, conduct initial screening, and ask eligible patients if they wish to participate.

Patients will be excluded if the screening call determines that they (1) do not plan to use VA primary care as their main source of diabetes care over the subsequent 12 months; (2) are not able to use a telephone to respond to twice monthly automated calls; (3) expect to have a > 1-month gap in care from their usual primary care site in the 12 months following enrollment (e.g., due to “snowbird” travel); (4) live in a nursing home or assisted living facility; (5) have significant cognitive impairment as measured by more than two of six possible errors on the Callahan six-item screener to identify cognitive impairment [44]; (6) need help with more than one of the six basic activities of daily living (ADLs) as measured by the Katz Index of Independence in ADLs [45]; (7) are not fluent in English; (8) have a life-limiting severe illness (end-stage renal disease (ESRD) requiring dialysis, chronic obstructive pulmonary disease (COPD) requiring oxygen, cancer undergoing active treatment, or heart failure with New York Heart Association (NYHC) III–IV symptoms); (9) are concurrently enrolled in another research study or VA program, at time of enrollment, that could conflict with CO-IMPACT’s protocol (e.g., another diabetes management intervention); (10) are concurrently enrolled in the VA Diabetes TeleHealth program; (11) are currently pregnant or planning to become pregnant in the next 12 months; or (12) are unable to identify an eligible adult family member or friend who is regularly involved in their health management or healthcare who consents to participate in the study.

In the final screening question, the patient will be asked “Do you have a family member or friend who gets involved with your healthcare in one of these ways...” followed by a list of specific support roles including help with medications, help with home glucose test results, tracking medical information, or coming to doctor’s appointments. In our preliminary studies, this question performed equally well at identifying highly involved and willing supporters as scoring multiple family members on the more complex Norbeck Social Support Questionnaire [46] research measure. Family member participants can either live with the patient or live separately. If the patient names someone, he/she will be asked if the family member meets the following criteria: (1) talks with the patient about the patient’s health at least twice monthly on average, (2) is at least 21 years old, (3) speaks English fluently, (4) lives in the USA, and (5) does not receive pay to provide care for the patient. If the potential family supporter is deemed ineligible, the patient will be asked to nominate another person to be assessed for eligibility.

Eligible patients who identify a potentially eligible family supporter will be encouraged to contact that family supporter to explain their interest in the study, and concurrently, the family member will be sent a letter that includes a study information sheet. After 1 week, a research assistant will call the potential family supporter to describe the study and assess interest and eligibility. Supporters will be excluded if, via the screening questions, they report that they (1) are younger than 21 years old; (2) have ever been told by a doctor that they have dementia, schizophrenia, or manic depression; or (3) receive pay for caring for the patient. They will also be excluded if they meet the following criteria, determined and defined in a similar manner to that described above in the patient screener: have significant cognitive impairment, need help with basic activities of daily living, or have a life-limiting severe illness. If the family member is interested and determined to be eligible, he/she will provide informed consent via phone.

#### Enrollment and randomization

Once the family supporter has provided verbal consent over the phone and is enrolled as the patient’s Care Partner, the patient will be asked to come to his/her usual primary care clinic for in-person informed consent and baseline study assessment (see the subsection Measures and analysis). During this enrollment visit, the research assistant will offer participants home glucometers and BP monitors if they would like them, but they are not required to have them. At the end of the visit, dyads will be randomly assigned to the intervention or the enhanced usual care group using an online randomizer that employs methods described by Pocock and Simon [47]. Randomization of patient-Care Partner dyads will be balanced across a single prognostic factor with two levels: living with Care Partner and living apart from Care Partner. Sampling rates will be equivalent (1:1) across both levels of the balancing variable. The minimization algorithm to balance treatment assignment uses the range function. The level of determinism will be set to 5 on a scale from 1 to 10 to increase balanced assignment of treatment condition while minimizing the predictability of treatment assignment.

#### CO-IMPACT intervention

The intervention period will last 12 months. The CO-IMPACT intervention will be delivered by a Dyad Engagement Coach (DEC) and an automated IVR telephone system (Fig. 3). The intervention content and design were developed from an earlier initial pilot version, conducted over 6 months with 19 patient-Care Partner pairs [48] who gave extensive feedback that was incorporated into the version used in this protocol.

##### Dyad Engagement Coach

The DEC will be an individual with a background in health education. The DEC will receive training from the study investigators in basic diabetes management; concepts underlying patient activation, motivational interviewing, action planning, and autonomy-supportive communication; and techniques to promote effective patient and supporter communication with clinicians.

##### Initial coaching session

Dyads will first attend one initial coaching session at their usual primary care clinic. Patients will be asked to attend this session in person, and Care Partners can attend in person or via speakerphone. Care Partners who participate by phone will be pre-mailed printed materials and guided to the intervention website during the session if possible. As shown in the initial session agenda (Table 1), this session serves to include supporters in the delivery of information that patients typically receive on their own, and it adds coaching in several skills that patients and family members do not typically receive in usual care. The content emphasizes roles the family can take in encouraging patients to make action plans and communicate effectively with their healthcare teams. Care Partner use of autonomy-supportive communication techniques with patients is a key focus (see Additional file 1 for a selection from the coaching script). Dyads will be encouraged to talk about diabetes weekly and given suggested conversation topics, including reviewing and adjusting patient action plans. During the session, participants will be encouraged to work together on an initial diabetes management goal and identify positive communication techniques they would like to use during their weekly conversations.

**Table 1:** Initial coaching session agenda

Review the patient’s diabetes complication risk status: last HbA1c, blood pressure, lipid levels, smoking status, and calculated UKPDS 5-year cardiac risk score	
Review the patient’s latest diabetes plan based on medical record progress notes and prescriptions	
Coaching on dyadic approach to goal-setting and action planning	
Coaching on use of positive and autonomy-supportive communication	
Structured talking points for biweekly patient-supporter discussions about diabetes and action planning	
Educate dyad about members of the patient’s PACT teamlet, their roles, and how to reach them	
Educate dyad about diabetes risk reduction programs available in PACT	
Techniques for effective and activated patient and supporter communication with patients’ medical providers	

##### Materials available to participants

After the initial session, patients and Care Partners will be able to review the guidelines and talking points discussed via a study website and a printed handbook (see Additional files 2 and 3 for excerpts from the handbook). These materials will include general information about diabetes management that is available in usual care, plus content on family supporter communication with patients and providers. Care Partner communication content is based on (1) content from theoretically based and effective research interventions, such as an autonomy-supportive communication intervention for family supporters of patients with heart failure [49], and (2) guidelines for caregiver-clinician communication, such as those produced by the National Family Caregivers Association.

##### Between-visit action planning prompted by automated telephone technology

Throughout the 12-month intervention, patient participants will receive IVR assessment calls once every 2 weeks. IVR is an automated technology that allows patients to report and receive information via a touchtone telephone. Calls can be placed at times convenient to the patient, and data are collected when patients answer pre-recorded voice prompts [50]. The goal of these calls is to prompt continued action planning and family supporter involvement between primary care visits. The core content is based on scripts developed by physicians, nurse educators, behavioral specialists, and experts in mobile health. Calls will last roughly 15 min, and content is guided by principles of patient activation and family supporter engagement. During each call, patients will be asked a series of questions to identify diabetes management concerns (see Table 2) that they could focus on in creating action plans. These include more than two fasting home glucose readings over 200 mg/dl or one under 80 mg/dl, two home SBP readings over 150 mmHg or any home SBP readings < 100 mmHg, bothersome medication side effects, running short on medication supply, or new foot problems. If any concerning health issues are identified during the call, following a patient empowerment approach, the patient will be asked whether he/she considers the identified issue important to address over the next 2 weeks. At the end of the call, the patient will be reminded to make an action plan to address one or two of these issues that he/she indicated as important. After each completed IVR call, the Care Partner will receive an automated structured email summarizing the call, with any identified health issues, which issues the patient considers important to address, and advice on how the Care Partner can support the patient with identified issues, including links to relevant content on the study website (see Additional file 4 for a sample email message). Emails will remind Care Partners to discuss diabetes care with the patient using the autonomy-supportive techniques discussed at the initial coaching session. The patient’s primary care team will receive an automated fax alert when patients indicate clinically urgent issues during their call (including ≥ 2 blood sugar readings < 70 mg/dl or ≥ 1 blood sugar reading > 300 mg/dl, or ≥ 2 SBP readings < 90 mmHg or ≥ 1 SBP reading > 170 mmHg).

**Table 2:** Topics covered in automated interactive phone calls

Inquire whether patient worked on an action plan based on the previous week’s call	
Patient illness severe enough to interfere with diabetes management	
Blood sugar levels: challenges to checking at home, low and high levels, and symptoms	
Blood pressure: challenges to checking at home, high and low readings, and symptoms	
Challenges to taking medications	
Readiness to make a plan to quit smoking (if applicable)	
New foot concerns	
Summary of call, inquiry about importance to patient of potential concerns identified	

##### Primary care visit preparation

The patient’s DEC will be alerted of the patient’s upcoming primary care visits via an automated weekly scan of VA appointment records. A qualifying visit will be an in-person visit to a primary care provider, nurse, or clinical pharmacist. Approximately 1 week before each qualifying visit, the DEC will conduct a preparation session with the patient via telephone. Using a visit preparation worksheet (see Additional file 5), the DEC will help patients identify any diabetes risk-related questions or concerns they would like to address during their visit, as well as diabetes-related information, such as home monitoring logs, they will bring to the visit. The DEC will invite patients to role-play, asking one or two questions most important to them. If a patient’s Care Partner is present with the patient at the time of the call, the DEC will suggest that the patient invite the Care Partner to participate in the call. Whether or not the Care Partner participates in the call, the Care Partner will be emailed or mailed a notification of the upcoming appointment that encourages them to use the visit preparation worksheet to note their questions and concerns for the patient’s visit. Both patients and Care Partners will be encouraged to share their questions and concerns with one another before the patient’s visit.

##### Primary care visit summaries

Within 1 week of a completed, qualifying primary care visit, the DEC will create a visit summary using an automated template programmed into the electronic medical record (EMR). The summary will include vital signs (including BP), recent lab results (HbA1c or lipid levels), medication prescriptions and changes, and a brief narrative section for diabetes-related issues discussed. The summary will be mailed to the patient, then 3 days later posted on the secure study website for Care Partners and patients to view or download. When a summary is posted online, the Care Partner will receive an email notification with a link to the summary.

##### Role of PACT clinical providers

Although the intervention is designed to ultimately be usable by medical teams, participants’ clinicians will not be directly asked to change their management of diabetes as part of the intervention. Before beginning enrollment, study team members will attend clinical staff meetings to provide an overview of the intervention and practical tips on interacting with family supporters who choose to call or attend visits with the patient. A written summary of the health information shared by the DEC at their initial session with the dyad will be placed in the patient’s medical record for their primary care team to view. This medical record note will also serve to alert the medical team to the Care Partner’s role and the fact that the patient has given permission to share personal health information with that supporter. The DEC will not change any components of the patient’s diabetes management plan (e.g., no changes to medications, ordered tests, or consults). If the patient asks the DEC about topics that are not included in intervention session protocols, the DEC will advise the patient to contact his/her nurse care manager for possible referral to an appropriate resource (such as diabetes education classes).

### Enhanced usual care (EUC)

Patients assigned to the EUC condition will receive usual care for diabetes at VA facilities that have implemented the VA PACT PCMH model. The fundamental components of VA PACT care for high-risk diabetes are co-management by a nurse or clinical pharmacist with the primary care provider, coupled with referral to VA chronic disease management support programs. The recruitment primary care sites have fully staffed and functional teams, defined roles and protocols for nurse care manager/clinical pharmacist management of diabetes, and available diabetes self-management classes, health psychology services, weight loss programs, and automated telehealth programs to monitor home sugar and BP results. Patients often receive visit summaries after their primary care appointments. Study staff will also provide patients in the EUC arm with general diabetes management information via handbook and website as well as home glucometers and BP monitors for patients who want them. EUC patients will not be precluded from involving family supporters in medical visits or VA health programs. Thus, the outcomes of the CO-IMPACT intervention will be compared to those of a patient offered a highly resourced and functioning PCMH, but without structured family support for self-management, action planning, and engagement in healthcare.

### Measures and analysis

#### Data sources

Data will be obtained via patient in-person and family supporter phone surveys at baseline and 12 months; patient and supporter 6 month surveys via telephone or mail; patient BP and laboratory measurements at baseline and 12 months; and patient pharmacy and clinical encounter EMR data from periods 12 months prior to baseline through 12 months beyond the intervention period. Data from recruitment and coaching session logs and IVR and website use will also be captured. See Additional file 6 for details on data management and security measures, safety monitoring, and reporting of adverse events. Assessments are described in detail below; assessment forms can be made available upon request.

#### Blinding

Baseline survey assessments will be conducted by a study staff person who is not aware of the participant’s study assignment, and all participant assessments will be conducted by a staff person not involved in the delivery of the intervention to the participant. Medical record data will be extracted and main outcomes analyses conducted by analysts blinded to group study assignment. Please see Additional file 7 for more details on this and other aspects of the study protocol.

#### Patient outcome measures

##### Health behaviors and behavioral determinants

The study’s main outcome measure will be the Patient Activation Measure-13 (PAM-13) [51]. The PAM-13 has been widely used to measure patient activation in longitudinal studies and in clinical trials as a primary outcome measure, and scores have been responsive to intervention [52]. The PAM-13 is reliable (Cronbach alpha 0.87) [53], and improvement in PAM-13 scores has been linked to improvement in self-management behavior [54]. A 4- to 6-point change in the PAM is considered clinically significant [52, 55–57]. We will also measure patient activation in medical visits with the Perceived Efficacy in Patient-Physician Interactions (PEPPI-5) [58]. Items include “I am confident in my ability…to get a doctor to answer all of my questions” and “to get a doctor to take my chief health concern seriously”. The PEPPI-5 has been validated against other self-efficacy and patient satisfaction scales, and it is reliable (Cronbach alpha 0.92) [59]. Table 3 lists other patient health behavior and behavioral determinant measures that will be assessed.

**Table 3:** Details on selected patient measures

Construct	Source	Instrument(s)	Baseline	6 mo.	12 mo.
Health behaviors and determinants					
Activation	Survey	Patient Activation Measure (PAM-13)	X	X	X
Activation in health encounters	Survey	Perceived Efficacy in Patient-Physician Interactions (PEPPI-5)	X	X	X
Diabetes self-efficacy	Survey	Stanford Chronic Disease Self-Efficacy Scale [72]	X		X
Diabetes distress	Survey	Problem Areas in Diabetes Scale [73]	X		X
Diabetes self-management behavior (self-monitoring, healthful eating, physical activity)	Survey	Summary of Diabetes Self-Care Activities [74]	X	X	X
Diabetes medication adherence	EMR × 12 months	Cumulative medication gaps < 20% [75]	X		X
Smoking status	Survey	Items from the World Health Organization’s Global Adult Tobacco Survey [76]	X	X	X
Physiologic and health outcomes					
5-Year cardiac event risk	survey + physiologic and lab testing	United Kingdom Prospective Diabetes Study (UKPDS) 5 year cardiac risk score	X		X
Glycemic control	Venous sample	HbA1c	X		X
Blood pressure	Direct measure	Systolic blood pressure, mean arterial pressure	X		X
Non-fasting lipid levels	Venous sample	Total cholesterol/HDL	X		X
Patient-supporter relationship and support quality					
Patient-supporter relationship quality	Survey	Relationship Rating Form - Respect Subscale [77]	X		X
Patient satisfaction with diabetes social support	Survey	Diabetes Care Profile -Support Subscale [78]	X	X	X
Supporter use of autonomy-supportive communication	Survey	Important Other Climate Questionnaire [79]	X		X
Potential moderators					
Time with diabetes	Survey		X		
Patient comorbidities	EMR × 12 months	Charlson Comorbidity Index [80]	X		
Health literacy	Survey	Brief Health Literacy Screen [81]	X		
Current PTSD symptoms	Survey	Primary Care PTSD Screen for DSM5 [82]	X		
Depression and anxiety	Survey	Patient Health Questionnaire-4 [83]	X		X

*EMR* electronic medical record, *HDL* high-density lipoprotein, *PTSD* post-traumatic stress disorder, *DSM* Diagnostic and Statistical Manual of Mental Health Disorders

##### Health risks

To address the effect of CO-IMPACT on patient health risks, our main measure will be the 5-year UKPDS Risk Engine [42]. This score estimates the risk of a coronary heart disease (CHD) event (fatal or non-fatal myocardial infarction, or sudden death) specifically among people with diabetes. The score components include factors we hypothesize could be improved by the intervention, including HbA1c, SBP, total cholesterol/high-density lipoprotein (HDL) cholesterol ratio, and smoking status. The score also includes age, sex, race/ethnicity, and length of time since diabetes diagnosis. Using a cardiac risk score to measure risk factor changes offers the advantages of quantifying the cumulative impact of changes in multiple risk factors and translating changes in physiologic parameters to a risk estimate that is meaningful to patients and policy makers. For similar reasons, cardiac risk scores have been successfully used as outcomes in multiple clinical trials [60–64], and the UKPDS Risk Engine has been validated in multiple populations [65].

HbA1c, lipid levels, BP, and smoking status will be analyzed independently as secondary health outcomes. We will measure via survey patients’ frequency of hypoglycemia and diabetes distress. Patients’ use of VA urgent care will be extracted from the EMR for the period 12 months prior to intervention start and during the 12-month study period, supplemented by patient report of non-VA urgent care.

##### Patient-supporter relationship and support quality

We will measure overall relationship quality for both patients and supporters (see Tables 3 and 4). Patient satisfaction with overall quality of diabetes support received and supporter use of autonomy-supportive communication will be assessed via patient survey. Supporters and patients will be surveyed about concerns about health privacy breaches.

**Table 4:** Details on selected supporter measures

Construct	Source	Instrument(s)	Baseline	6 mo.	12 mo.
Behaviors and determinants					
Supporter self-efficacy for helping patient with diabetes mellitus care	Survey	Adapted Stanford Chronic Disease Self-Efficacy Scale [72]	X	X	X
Health and relationship outcomes					
Caregiver burden	Survey	Caregiver Strain Index [66]	X		X
Supporter distress about patient’s diabetes	Survey	Adapted Problem Areas in Diabetes Scale [73]	X	X	X
		Adapted Fear of Hypoglycemia - Worry Subscale [84]	X		X
Patient-supporter relationship quality	Survey	Relationship Rating Form - Respect Subscale [77]	X		X
Potential moderators					
Depression and anxiety	Survey	Patient Health Questionnaire-4 [83]	X		X

##### Patient-provider relationship and patient satisfaction with VA healthcare

We will measure patient satisfaction with VA primary care and their primary care provider, using questions from the VA Consumer Assessment of Healthcare Providers and Systems (CAHPS)-PCMH. We will also ask about patient satisfaction with VA engagement of their family supporters.

#### Family supporter outcomes

We will assess family supporter roles (e.g., helping to track patient medication use at home) via surveys at baseline, 6, and12 months. Family supporters’ self-efficacy for helping patients with.

diabetes, supporter distress about the patient’s diabetes, and supporter distress about patient hypoglycemia will be measured with adaptations from similar validated patient measures (Table 4). Caregiving burden will be assessed with the reliable and validated Multidimensional Caregiver Strain Index [66].

#### Patient and supporter moderators of effect

Theoretical patient moderators of intervention effects that will be measured include (Table 3) sociodemographics (sex, age, education), baseline diabetes medication regimen, distance from VA site, comorbidities, health literacy level, and comorbid depressive symptoms [67]. Additional moderators include whether the patient and supporter live together, whether the supporter has diabetes, supporter depressive symptoms, baseline patient-supporter and patient-physician relationship quality, and whether family supporters attend patient visits in person.

#### Intervention and control processes

Because the study is designed to determine whether adding an incremental amount of attention towards patients’ family supporters leads to benefits to patients’ engagement, health behaviors, and health outcomes, the study will track rates of primary care and DEC visits, phone calls, and letters; visit summaries; and use of telehealth monitoring to evaluate differences in staff attention between participant groups. Specifically, we will record the frequency of each type of DEC contact with intervention-assigned participants and the DEC time spent in preparation and execution of each contact. We will automatically capture the outcomes of all IVR call attempts and the number of visits to and downloads from the study website. For participants in both arms, we will capture via EMR the number of completed primary care provider, nurse, and clinical pharmacist encounters, occurring in person or by phone. We will ask participants via survey whether they received visit summaries after in-person primary care visits in both the intervention and control conditions. We will tally consults entered by primary care teams to diabetes risk-related programs as well as patient (via EMR) and supporter (via survey) rates of attendance. Finally, we will ask all patients and supporters about the frequency of general discussions about diabetes, pre-visit preparation discussions, and post-visit debriefing.

#### Intervention fidelity

A predetermined sequence (the first 10, then 10% of the remaining by random number generation) of DEC initial and telephone sessions will be recorded for review by the study Principal Investigator (PI) and key investigators, along with DEC-created documents. A checklist form will facilitate standard fidelity reviews. Patient appointment and IVR call records will be monitored regularly by study staff for level of missed contact opportunities.

#### Retention and follow-up

Previous studies have found that, once recruited, dyads have better study retention than patients participating alone [37, 68]. Nevertheless, we will use several strategies to maintain high retention, such as patient incentives that cover cost of transportation for in-person assessments at VA sites and staff travel to patients’ local VA sites. We will offer patients the option to coordinate in-person assessments with VA appointments. We will offer family supporters phone and Internet options for all study and intervention procedures. For patients receiving IVR calls, call completions are monitored, and if patients miss their first call or three sequential calls, the DEC calls to follow up, troubleshoot any issues preventing call completion, and encourage call completion. If after several attempts we cannot complete a patient assessment in person, we will ask the participant to complete the survey assessment over the phone, with an option for a short version of our assessment that prioritizes key measures: the Patient Activation Measure (PAM-13), smoking status, diabetes self-management behaviors (taking medications, exercise, healthful eating), and daily insulin doses.

### Analysis plan

We will follow international guidelines for analysis and reporting of clinical trials [69]. We will examine baseline data for prognostically important differences across the two study groups, such as patients’ age, race, comorbidities, and baseline use of services. Although we do not anticipate any imbalances, any baseline differences between experimental arms will be included as covariates in analyses comparing outcomes. Missing data will be imputed for non-outcome measures, using multiple imputation methods. If we find baseline variables to be associated with the loss to follow-up, we will include those baseline variables as covariates in models evaluating the intervention effect.

#### Unit of analysis and sample size calculation

Our main aims are to evaluate effects at the patient level. Our sample size calculations are based on our primary outcome of patient activation, measured by the PAM-13. Assuming that the PAM-13 was highly correlated between baseline and 1 year (*r* = .70), we calculated our sample size to provide a minimum of 80% power to detect a between-group difference in PAM-13 change of 4.0, with a standard deviation of change of 13, and a two-tailed alpha of 0.05. To achieve 80% power, a minimum of 102 patients is needed in each group, for a total sample size of 204. To allow for 15% attrition, we will enroll 120 patients in each group, for a total of 240 patients. For our second aim, assuming the underlying correlation between UKPDS at baseline and 1 year later is .90, our sample size of 102 per group will provide more than 80% power for detecting between-group differences in predicted cardiac risk of 2.0% (standard deviation (SD) = 12), which can be considered clinically significant on a population level.

#### Primary analyses

All main analyses will be conducted using intention-to-treat principles. Main analyses will be performed using hierarchical linear models (HLMs) with scaled PAM scores (at baseline and 1 year) and UKPDS scores (baseline and 1 year) respectively as the outcomes. HLMs, or mixed models, incorporate both fixed and random effects. Fixed effects include treatment group, time, and whether the Care Partner and participant lived together; all are dichotomous predictors. Patient-level random effects will be included in the model to account for correlations between patients’ repeated measures over time.

#### Secondary analyses

As a supplementary analysis, we will analyze differences in PAM score by interacting with four baseline PAM strata, as a priori defined by the PAM scale developer. As another supplemental analysis, the curvilinear trajectory of the PAM-13 over time will be tested with growth curve modeling using the PAM-13 score at 6 months in addition to the score at baseline and at 1 year. Further, random effects models will be used to examine differences in this trajectory based on treatment group. Selected individual components of the UKPDS will be analyzed independently as secondary health outcomes using the same modeling strategy as outlined for the primary analyses above: HbA1c, SBP, non-HDL cholesterol, and smoking status.

#### Mediators and moderators of intervention effect

We will use multivariable regression models to examine potential mediators and moderators of intervention effects. We will introduce potential mediators to models linking intervention condition to outcomes, examining changes in the magnitude of the relationship between the intervention and the outcomes before and after the covariates are introduced. We will also use the Preacher and Hayes bootstrapping method to examine potential mediators to determine whether the mediation effect is significant [70]. This is a non-parametric method that can be used when the outcome violates assumptions of normality. Potential mediators are specified in our theoretical model (Fig. 1) and include an index of family supporter engagement in the intervention, composed of measures of supporter participation in intervention sessions and reported use of pre-visit preparation and debriefing tools. Analyses of potential moderators will use standard approaches to evaluate interactions between these covariates and the intervention, which will include plotting regression lines for high and low values of the moderator variable using Stata routines [43]. Independent variables and moderators will be centered before testing interactions, so that multicollinearity between first-order and higher-order terms will be minimized.

#### Process evaluation

We will use the RE-AIM framework [71] to guide this analysis. To analyze the potential reach of the intervention, we will calculate the proportion of patients with diabetes who meet inclusion criteria and compare characteristics of eligible and non-eligible dyads. Effectiveness will be measured via our main outcomes and differences in outcomes among key patient groups as described above. We will evaluate adoption by examining the characteristics of patients and supporters who decline enrollment and their reasons for declining. We will also examine retention/dropout from the study and reasons, length/frequency of DEC sessions, percentage of potential DEC sessions completed, and IVR call adherence (percentage of attempted calls completed, number of weeks adherent to calls).

## Discussion

The CO-IMPACT intervention takes a unique approach to meeting the self-management support needs of high-risk patients by empowering a family supporter to help the patient with key self-management and healthcare navigation skills. The intervention will provide a structured approach for clinical providers to engage family supporters in patient self-management support, using delivery methods designed to put minimal strain on clinicians by maximizing automated and technologically delivered information sharing with family supporters. CO-IMPACT is innovative in its approach to increase family supporter effectiveness through (1) training in effective ways to communicate with patients about their healthcare, (2) training in effective ways to support patient engagement in their healthcare, and (3) providing actionable and patient-specific health information. The information-sharing and core skills are meant to enable patients and family supporters to better take action on recommendations made by their healthcare providers or diabetes educators outside the CO-IMPACT program, and can be used over time to respond to changing patient health situations and needs.

This study comparing CO-IMPACT to enhanced usual care will allow us to assess whether providing an incremental amount of attention and training to family supporters results in valuable improvements in patient health for patients who are at high risk for poor outcomes. The study emphasizes outcomes that are patient-centered while also evaluating impacts on patient-family and patient-provider relationships. In addition, the techniques developed in conducting this study will inform health system efforts to screen for the presence of family members and other caregivers involved in patient healthcare and to assess family-related needs and outcomes relevant to care quality and patient satisfaction.

CO-IMPACT is designed to tap into and harness the potential of the large pool of family supporters to effectively improve self-management and healthcare engagement among patients with complex healthcare needs; these family supporters include those family members who live at a distance from the patient but are still regularly involved in the patient’s healthcare. This study will move beyond the status quo by focusing on families as the context for, and as key players in, diabetes “self”-management. If family members can more effectively support healthcare, this would represent a novel source of support that could be sustained over the long term needed to meaningfully reduce diabetes complications. In addition, because CO-IMPACT focuses on self-management and patient communication skills that could be applied across health conditions, the lessons learned in this study could be easily applied to patients with other health conditions or patients with multiple chronic conditions. If successful, we expect this study to produce an evidence-based protocol and tools that engage patients with high-risk diabetes and their family supporters in healthcare to help patients achieve improved diabetes outcomes. Study results will be disseminated through multiple communication strategies including academic conferences and publications, professional societies such as healthcare provider and diabetes educator organizations, healthcare system operations leaders, and patient advocacy groups focused on diabetes and veterans’ health. A summary of the results will also be mailed directly to our participants. More generally, this study will contribute to the growing body of knowledge on how healthcare providers can most effectively engage family supporters and caregivers in patients’ care to optimize health management and outcomes.

## Trial status

This protocol is version number 6, approved by the Institutional Review Board (IRB) on September 11, 2016. Recruitment began November 30, 2016, and recruitment is expected to complete in June 2018.

## Additional files

Additional file 1: Section of initial coaching session script. (PDF 623 kb)

Additional file 2: Sample from handbook on patient-Care Partner teamwork. (PDF 1251 kb)

Additional file 3: Sample from handbook on teamwork with HCP. (PDF 676 kb)

Additional file 4: Sample email message to Care Partner. (PDF 270 kb)

Additional file 5: Visit planning worksheet. (PDF 209 kb)

Additional file 6: Data management and security, safety monitoring, and adverse events. (DOCX 18 kb)

Additional file 7: SPIRIT checklist. (DOCX 67 kb)

## Abbreviations

ADL: Activity of daily living
BMI: Body mass index
BP: Blood pressure
CAHPS: VA Consumer Assessment of Healthcare Providers and Systems
CDW: Corporate Data Warehouse
CHD: Coronary heart disease
CO-IMPACT: Caring Others Increasing EnageMent in Patient Aligned Care Teams
COPD: Chronic obstructive pulmonary disease
DEC: Dyad Engagement Coach
DSM: Diagnostic and Statistical Manual of Mental Health Disorders
EMR: Electronic medical record
ESRD: End-stage renal isease
EUC: Enhanced usual care
HbA1c: Hemoglobin A1c
HDL: High-density lipoprotein
HLM: Hierarchical linear model
IVR: Interactive voice response (telephone system)
PACT: Patient Aligned Care Teams, the VHA’s patient-centered medical home
PAM-13: Patient Activation Measure-13
PCMH: Patient-centered medical home
PEPPI-5: Perceived Efficacy in Patient-Physician Interactions, 5 item
PTSD: Post-traumatic stress disorder
SBP: Systolic blood pressure
SD: Standard deviation
SM: Self-Management
UKPDS: United Kingdom Prospective Diabetes Study
VA: Veterans Administration
VHA: Veterans Health Administration
