# Supplementary material for: Engaging family supporters of adult patients with diabetes to improve clinical and patient-centered outcomes: study protocol for a randomized controlled trial
Source: Trials. 2018 Jul 24;19:394. doi: 10.1186/s13063-018-2785-2 (PMC6057090; doi:10.1186/s13063-018-2785-2)
Supplement: Supplementary file 1 — Section of initial coaching session script. (PDF 623 kb) [file 13063_2018_2785_MOESM1_ESM.pdf]

Initial Coaching Session: Selections from Dyad Coach's Script

<table>
<tr><th colspan="2">Strategy #2 TECHNIQUES FOR WORKING TOGETHER WITH CARE PARTNERS</th></tr>
<tr><td>
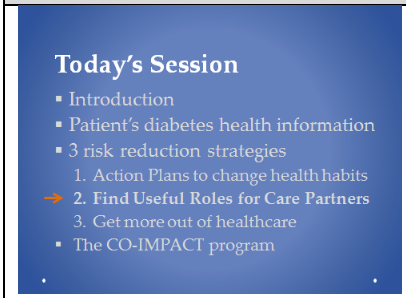

</td><td>

Intro

Let's move on to strategy #2, finding useful roles for Care Partners.

</td></tr>
<tr><td>
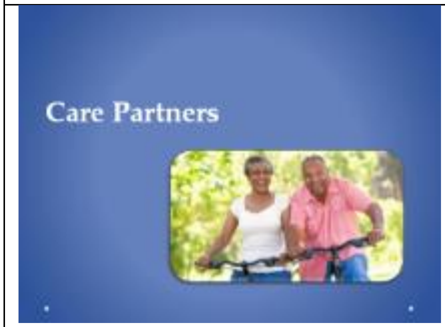

</td><td>

What do you know about how having a support person affects your health? For most patients we work with, patients who have a family member or friend supporting them in working on their health goals are more successful and end up healthier than those who go it alone.

But there are more and less helpful ways for family members to get involved. Now we will talk about specific Ways that Work when Care Partners and Veterans work together to make positive health changes.

</td></tr>
<tr><td>
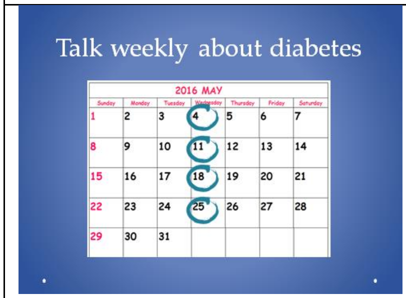

</td><td>

Weekly Talks

In order to stay on top of what's happening with [patient's] health, how often do you think you need to talk with each other? For how long?

Our experience has been that patients that can find 10-15 minutes a week is the best way to not let health issues slip by.

When do you think you would have time to do that?

It may help to establish a routine, like talking after dinner every Wednesday, or talking on the phone every Saturday morning.

- *Initiate discussion about when they might do this. Many family members already have a routine that they visit or call certain days of the week. So the pair can plan that the first 10 minutes of their weekly phone call will be about health, then they can move on to other things.*

</td></tr>
</table>

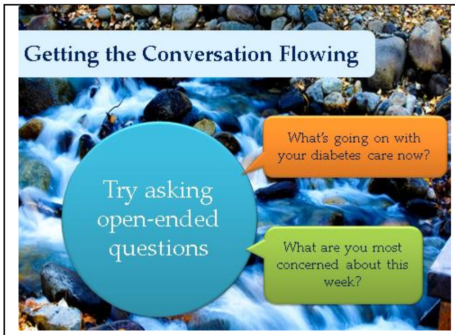

Sometimes family members want to talk to each other about health, but it's hard to know what to talk about.

(or: have you ever wished that you could talk about your (or the patient's) health as easily as you may talk about other things, like a TV show you like?)

I have some suggestions that might help you start the conversation.

<u>#1 Open-ended questions</u> will give Care Partners much more information than questions that ask for a yes or no answer. What would you say if I asked you

- "Did you take your medications last week?"
- "Were your sugars high this week?"
- ➢ *Give patient a chance to answer "yes" or "no"*

Instead, try asking questions like:
· "What's going on with your diabetes care now?"
· "What are you most concerned about this week?
(*If time, could let patient actually answer to show that more info naturally comes out*)
(if time, could ask patient "How would you like your CP to start these weekly conversations?')

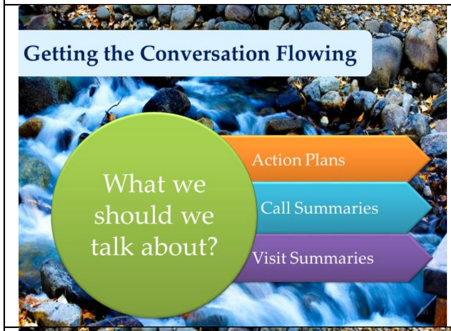

<u>#2 What should we talk about?</u>
If you aren't sure what to talk about, we have a couple of ideas if you'd like to hear.

You can start by just talking about how diabetes care is going. Or ask about these: 1) call summaries, 2) visit summaries or 3) the patient partner's last action plan

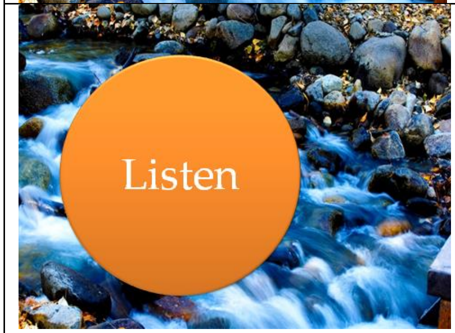

<u>#3 Then Listen! This is one of the most helpful things a Care Partners can do to help is to LISTEN</u>. It may not seem like much, but sometimes just being able to talk with another person (like you) can help a person with diabetes think through what they want to do.

<table>
<tr>
<td>
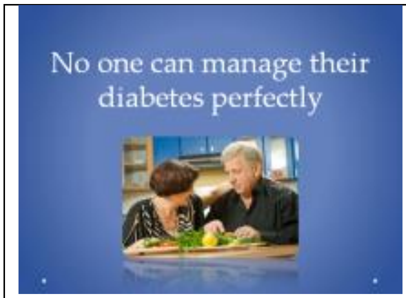

</td>
<td>
<u>No one can manage their diabetes perfectly</u>. Everyone misses medications or eats things they probably shouldn’t from time to time. Sometimes, people aren’t always honest with the Care Partners about how their diabetes is really going. Why might that be?

Some people have said…

Afraid they’ll be criticized or judged
Are embarrassed
Avoiding doing hard things

</td>
</tr>
<tr>
<td>
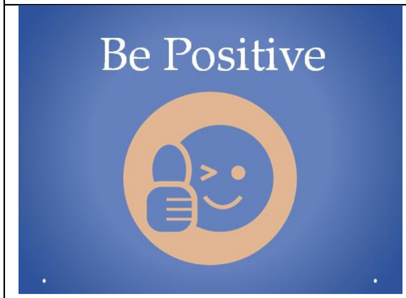

</td>
<td>
<u>Staying positive</u>
(to Care Partner) Patients often find it easier to be honest if their Care Partners stay positive. Staying positive means avoiding criticism and <u>congratulating</u> your partner for <u>trying to change</u>, even if they are not successful. Encourage them to keep trying, and ask how you can help them.

(to patient) Try to be as honest as possible with your Care Partner about how you are doing. They can give the most helpful advice if they know what’s really going on – what’s going well and what’s not going so well. Even if you don’t always agree with advice your Care Partner gives, try to <u>listen carefully and be respectful</u>

Also, let your Care Partner know when they do something that feels particularly helpful. For example, “Thanks for asking me to go for a walk, that was just what I needed to get up and get going”

(optional) Can you think of a time when your partner was positive and it really helped you think through a problem?
</td>
</tr>
<tr>
<td>
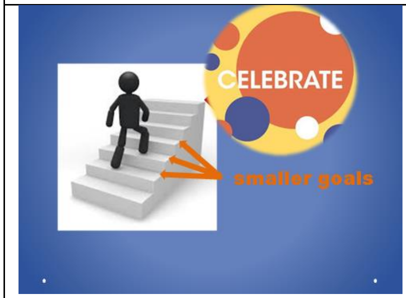

</td>
<td>
(to both) Acknowledge or even <u>celebrate</u> the changes we and others do make, no matter how small. Savoring those little successes can really help keep up motivation.

Like we talked about before those small steps can add up to significant changes.

[Can suggest to CP that they can point out positive changes the patient has made because sometimes people don’t notice the changes they make]
</td>
</tr>
</table>
