# Supplementary material for: Engaging family supporters of adult patients with diabetes to improve clinical and patient-centered outcomes: study protocol for a randomized controlled trial
Source: Trials. 2018 Jul 24;19:394. doi: 10.1186/s13063-018-2785-2 (PMC6057090; doi:10.1186/s13063-018-2785-2)
Supplement: Supplementary file 2 — Sample from handbook on patient-Care Partner teamwork. (PDF 1251 kb) [file 13063_2018_2785_MOESM2_ESM.pdf]

Patient - Care Partner Teamwork

Guidelines for weekly talks

It is helpful if the two of you talk regularly about the patient's diabetes care, either on the phone or in person. We recommend that you talk once a week so that you can go over the report from the patient's most recent CO-IMPACT automated call and any action plans the patient tried in the last week. Below are some guidelines for your weekly talks.

1. Try talking about diabetes care on the same day of the week and at the same time of day. This will help you both establish a routine.

2. Try to focus on the patient's health care for at least 10 minutes.

3. Over the course of a week, although you may talk about other family matters, what's in the news, or other interests you share, be sure to reserve a regular time to review the recent diabetes call summary or visit summary.

4.. Make sure that you don't feel rushed. Try to focus your full attention on the conversation without being distracted by other things such as children, work, or television.
Some people find that making a written list of things you want to cover before the conversation can be helpful.

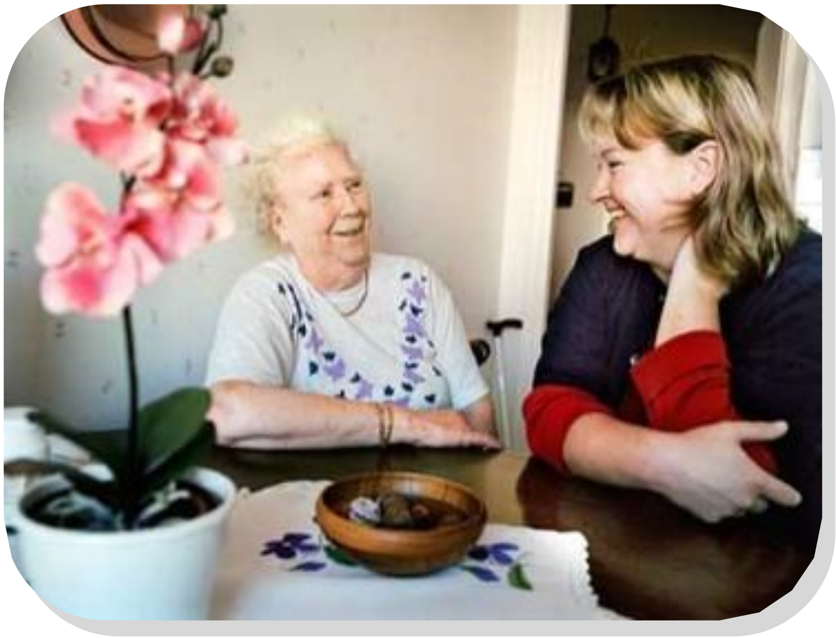

Tips for Patient Partners: Talking with Care Partners About Health

- **These conversations are meant to focus on how YOU are doing with your diabetes self-care.**
  Even if it feels a little uncomfortable rst, it is important to focus on your own health rather than other things that you both might like to discuss. After you've reviewed your health assessments and talked about your diabetes self-care, you can move on to other topics. Remember, your Care Partner has agreed that this is important.

- **Remember that your Care Partner is trying to help.**
  Even if you don't always agree with advice your Care Partner gives, try to listen carefully and be respectful.

- **No one can manage their diabetes perfectly.**
  Everyone misses medications or eats things they probably shouldn't from time to time. It is very important to be as honest as possible with your Care Partner about how you are doing. Even if you are a little embarrassed or worried that they'll be disappointed, try letting your Care Partner know about the things that might not be going so well. Your Care Partner is there to help!

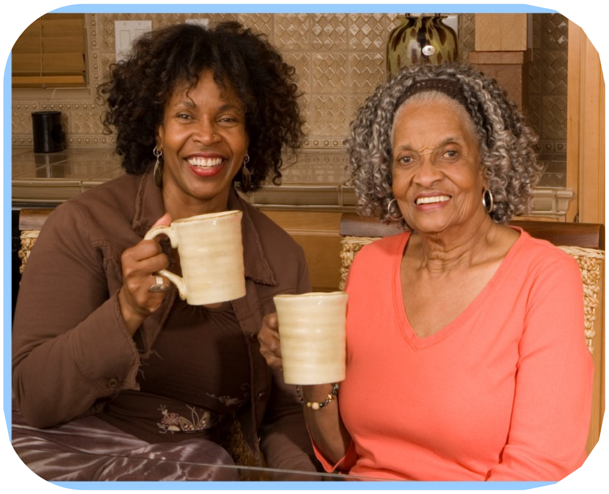

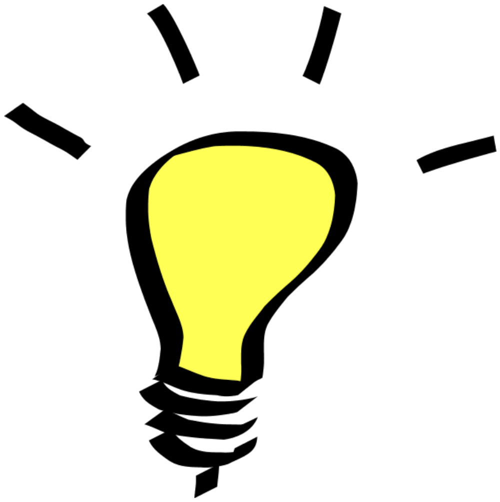

- **Do your best to come up with ideas and solutions to problems on your own, and then discuss them with your Care Partner.**
  YOU are the best person to know what will and what won't work, but sometimes it can be helpful to talk through the options with someone. If you don't know what to do, don't worry. You and your Care Partner can work on it together.

- **Agree on action plans with your Care Partner and try them out.**
  Try to be honest if you have doubts about your ability to follow-through on ways to meet your goals and come to an agreement with your Care Partner about what might be best to try. If things don't work out, you can discuss why it didn't work the next time that you talk and make a change of plans.

See the *Action Planning* section in this handbook for information, tips and examples about action planning.

Tips for Care Partners: Getting the Conversation Flowing

Open-ended questions will give you much more information than yes/no questions. Try asking questions like:

- "What's going on with your diabetes care now?"
- "What are you most concerned about this week?

Reflective comments help someone know that you are really trying to understand what they're saying and encourage them to tell you more. You are really focusing on them and trying to put yourself in 'their shoes'. Reflecting your partner's statements doesn't mean you agree, but it gives you both a chance to make sure you are understanding what the patient is trying to tell you. You might try using phrases like:

- "It sounds to me like you're feeling…."
- "Let me know if I've got this right, you tried to…."
- "It seems like you thought that it was really difficult to…"

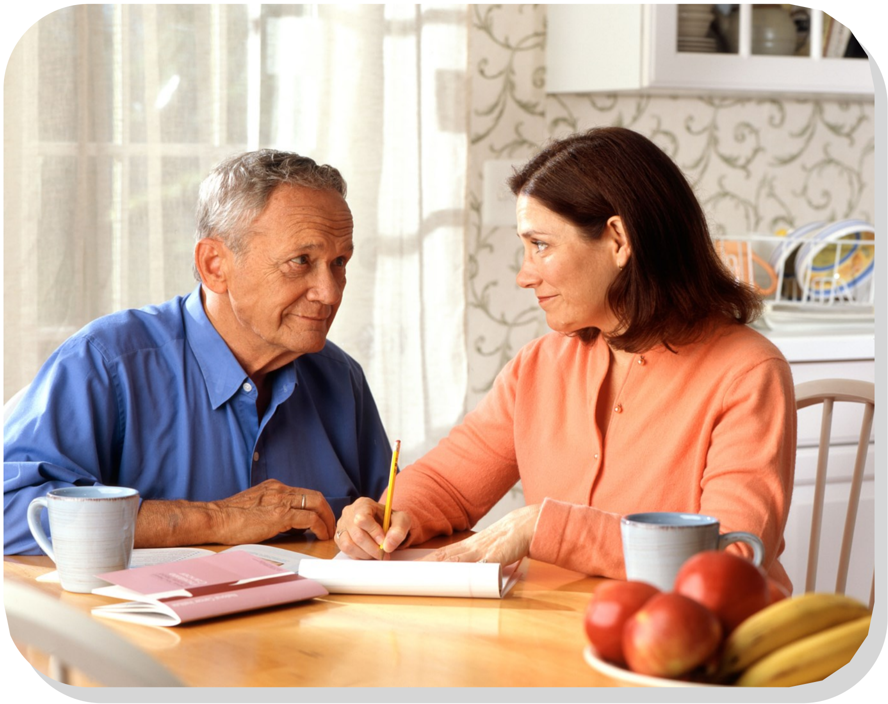

Tips for Care Partners: For a Positive and Helpful Conversation

One of the best things you can do to help is to LISTEN. It may not seem like much, but listening to your partner can often help them to be clear about what they are feeling. Sometimes just being able to talk with another person (like you) can help a person with diabetes think through what they need to do.

Listening to your partner can help them see what steps they can take to improve their health, and it can help you realize how you might help them.

It's sometimes easy to be critical if your partner doesn't follow through with their plan, or doesn't change in ways that would help them manage their diabetes better. However, if you criticize, judge, or ridicule someone with a chronic illness, that can make them feel discouraged. Having diabetes can be frustrating, so it's important that you do your best to encourage your partner whenever you can.

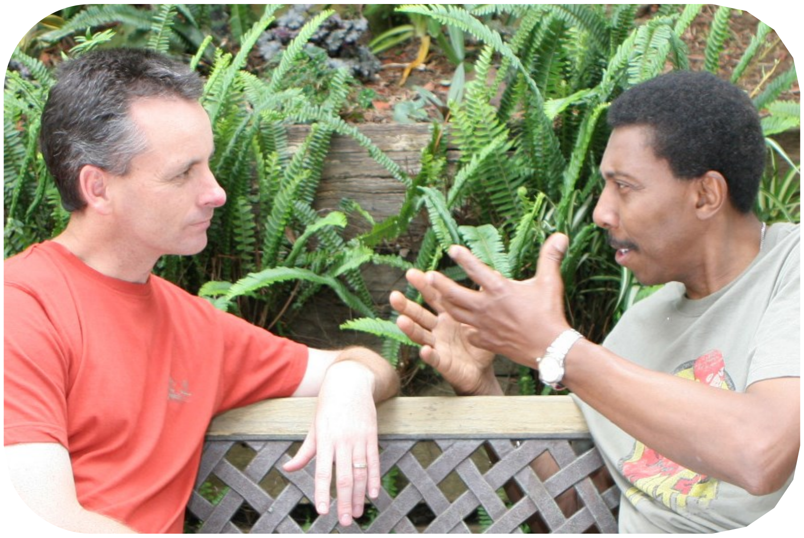

Try to accept that your partner is the one responsible for their diabetes care choices and actions. People tend to be more successful at living healthier when they feel that their motivation comes from within themselves, and they are free to choose what they feel are the best ways to stay healthy.

DO:

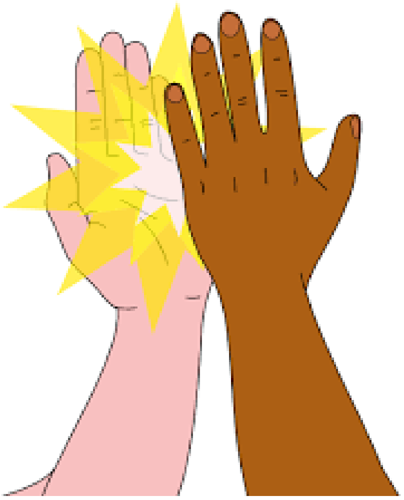

- Ask for your partner's ideas for ways to solve problems before offering your own.
- Offer your ideas as choices, instead of telling your partner what to do.
- Congratulate your partner for trying to change, even if they are not successful.
- Congratulate them for the changes they do make, no matter how small.
- Acknowledge that managing diabetes can be frustrating when they are having difficulties.
- Try to really understand what they are feeling and going through before offering any suggestions of things they might do.

DON'T:

- Don't criticize or be judgmental – your role is to listen, to encourage, to be available, to help remember, to troubleshoot, to be regular and consistent, and simply to be involved!
- Don't "take over." Sometimes you may feel that it would be easier if you did things yourself, rather than allowing your partner to take action. But this won't help your patient-partner in the long run. The best strategy is to be a helper, not the boss.
- Don't tell your partner what to do – make suggestions, but ultimately it's their decision.

Examples of Supportive Care Partner Comments

DO express empathy and concern

- “I appreciate how hard it is for you to think about walking again today.”

DO offer choices

- “Would you like me to help plan the menu for this week?”
- “What are some ways that you think might help you remember to take that evening dose of medicine?

DO acknowledge accomplishments, even small ones

- “It’s great that you tried. Remember, even small steps can get you where you need to be over time.”

DO help with problem solving

- “This is the second time that you have run out of that diabetes medication. What ideas do you have about how to keep that from happening again?”

When you suggest changes, explain why

- “Remember, the doctor explained that sugars going up over a short time means that you may be getting sick. When your sugars are that high we need to call the doctor’s office to let them know. They may tell you to take extra insulin or check your sugars more often until they are back to normal.”

AVOID language that is controlling, criticizing, or guilt provoking :

- Say: “Your doctor said that checking your sugar every day is an important way to keep track of your health. How are you feeling about that?”
  (NOT: “You know the doctor told you to check your sugar every day! I don’t know why you won’t do what he says!”)
