# Supplementary material for: Engaging family supporters of adult patients with diabetes to improve clinical and patient-centered outcomes: study protocol for a randomized controlled trial
Source: Trials. 2018 Jul 24;19:394. doi: 10.1186/s13063-018-2785-2 (PMC6057090; doi:10.1186/s13063-018-2785-2)
Supplement: Supplementary file 3 — Sample from handbook on teamwork with HCP. (PDF 676 kb) [file 13063_2018_2785_MOESM3_ESM.pdf]

Getting the Most Out of Appointments: For Care Partners

Not all Care Partners accompany their Patient Partners to their medical appointments. If your Patient Partner wants you to attend, and it fits into your schedule, consider going to at least one of their VA primary care appointments.

Patients who are accompanied to medical visits by a trusted supporter often communicate better with their doctors and nurses. They are able to get more of their questions answered and remember more of the information they hear.

Also, when you attend your partner's appointment, you can get to know your partner's doctors and nurses. That might make it easier if you need to contact them in between your Patient Partner's appointments.

Some tips for Care Partners attending medical appointments:

- **Educate yourself about your loved one's condition**. Use the Internet as a tool, but try to stick with reliable sources. You can ask doctors or nurses which websites they respect.
- **Write it down**. Have everything that's on your mind written down before you talk with the doctor. The more organized you are, the more help you can get. You can discuss with your Patient Partner what you both think should be on the visit planning worksheet you can find in the back of this handbook.

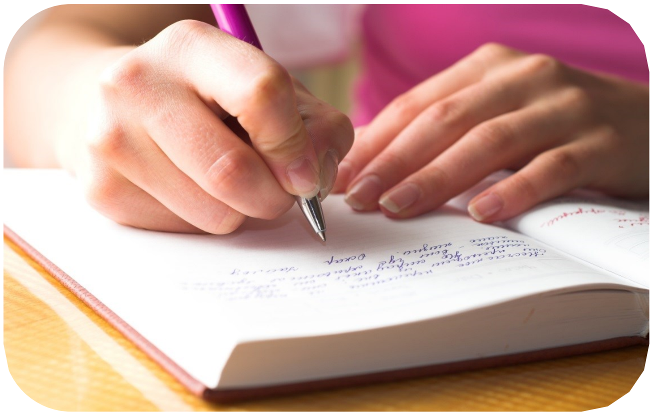

- **Let your Patient Partner take the lead when possible.** You can prompt your Patient Partner to use the question list that the two of you prepared before your appointment.
- **It is best to ask questions at the beginning** of the appointment. Talk about the most important concerns first. Be brief and stick to the point.
- **After your Patient Partner is finished** describing a problem or concern, you can fill in extra details.
- **After your Patient Partner has finished** asking their questions, you can add any of your own.
- **If you disagree with your Patient Partner, try to point it out in a neutral and specific way**. For example, "Hmmm. He used to check his sugar three times per day but when I look at his sugar log I only see numbers written down once per day." Don't overgeneralize or make accusations. (*NOT* "He is so lazy. He never tries to take care of his health.") Staying positive is helpful to everyone.
- **If the doctor or nurse gives recommendations, make sure you and your Patient Partner understand.** Don't hesitate to ask when the instructions are not clear. Make sure you understand what the next steps are.

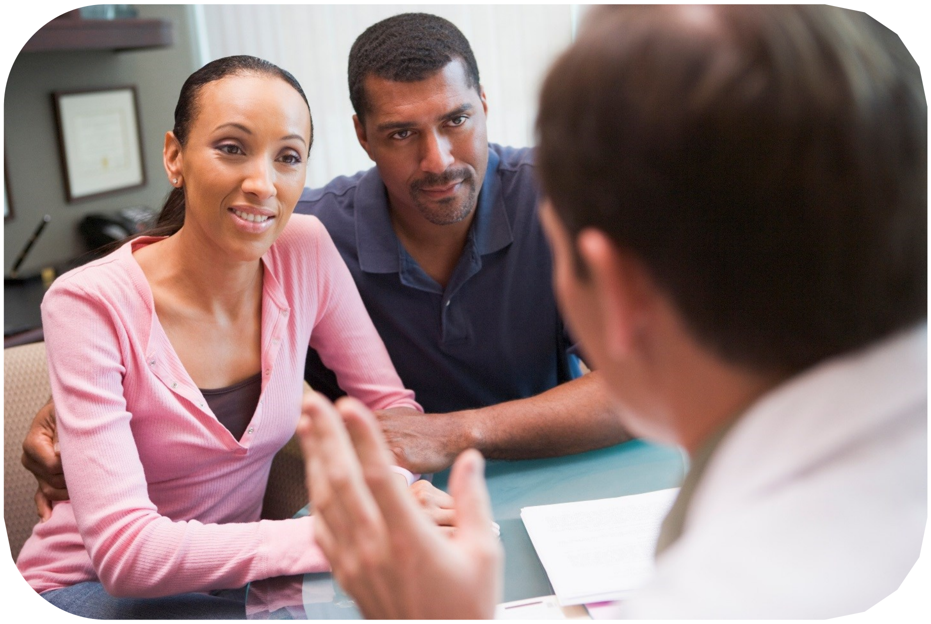

- **Help the doctors and nurses understand your role**. If there are certain parts of health care you help with, or certain health information you keep track of, tell the doctors and nurses. Make sure the doctor tells you all the information related to what you do. Ask them to keep you informed even when you can't come to the patient's medical visits.
- **Appoint one family member** as the main family contact with healthcare professionals when possible. This will avoid confusion and save time for everyone.
- **Ask about other resources**. The primary care staff can often point you to health programs or resources that might help your Patient Partner.

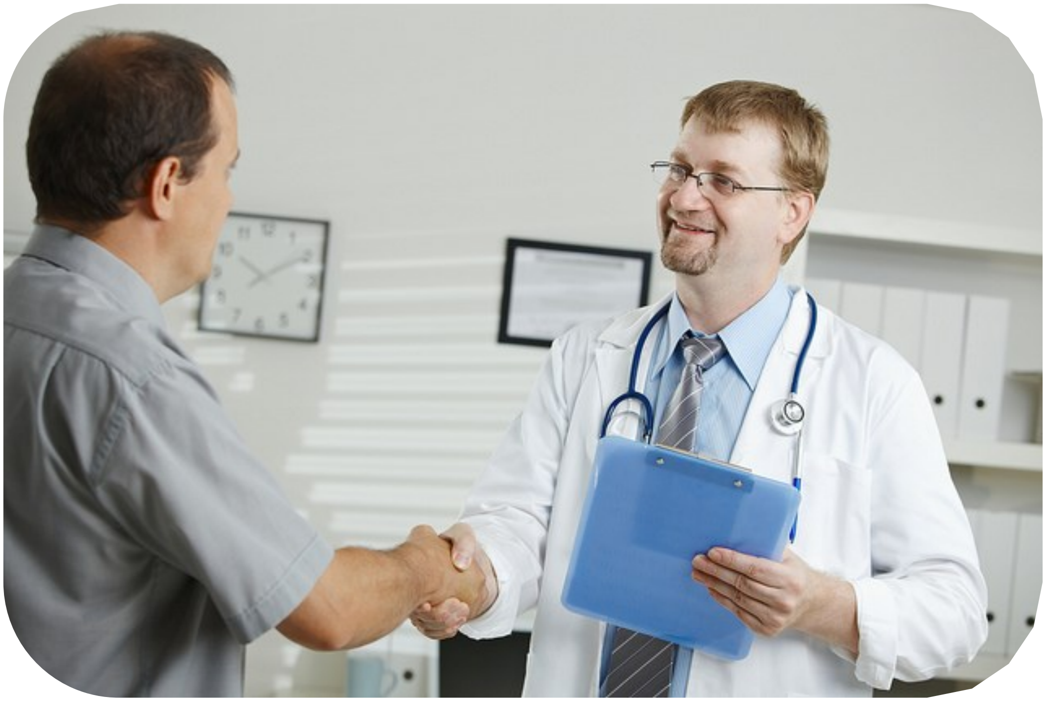

Between Appointments: For Care Partners

After an appointment

- Help and encourage your Patient Partner to make plans for how to follow their doctor's or nurse's recommendations.
- Discuss the Visit Summary that you and your partner receive.
- Talk to your Patient Partner about making appointments to have tests done or to see specialists if your partner needs to.

You and your partner should plan to contact your partner's primary care team if your partner:

- has problems following, or does not understand, the doctor or nurse's instructions.
- does not receive test results within a week of the test date. Do not assume that no news is good news.
- does not understand test results.
- experiences any side effects or other problems with their medicines.
- has symptoms that get worse (or do not get better).
- receives any new prescriptions from another doctor or starts taking any over-the-counter medicines.
- has any questions about their self-care regimen.

Patients who talk with or call their primary care team tend to be happier with their care and have better medical results. You should encourage your Patient Partner to contact their primary care team themselves whenever possible. However, if your Patient Partner feels that you are the best person to call, that is OK too. If you call, be sure to write down what the doctor or nurse says, and share it with your Patient Partner as soon as possible.

Available for Use with Permission from the Authors
