# Supplementary material for: Engaging family supporters of adult patients with diabetes to improve clinical and patient-centered outcomes: study protocol for a randomized controlled trial
Source: Trials. 2018 Jul 24;19:394. doi: 10.1186/s13063-018-2785-2 (PMC6057090; doi:10.1186/s13063-018-2785-2)
Supplement: Supplementary file 4 — Sample email message to Care Partner. (PDF 270 kb) [file 13063_2018_2785_MOESM4_ESM.pdf]

Your partner completed their most recent CO-IMPACT Care Partner telephone call on September 1, 2017 at 3:17 pm. Read below for a summary of:

- any urgent issues
- updates on regular monitoring
- any issues that your partner may have reported

*****Potentially Urgent Concern(s)*****

There are no potentially urgent concerns to report.

Update on Action Plans

STATUS OF ACTION PLANS FROM LAST CALL: Your partner reported they spent time working on their diabetes action plans they made after their last automated phone call.

WHAT IT MEANS: They can be congratulated! However, there may be some action plans they still want to work on.

HOW YOU CAN HELP:

- Ask how well their action plan worked to address their diabetes concern.
- If they changed their diabetes or health routine, ask if there is a way you can help them keep up the new routine.
- If they were not able to make the changes they wanted to make, ask what they learned from trying to make the change. Then discuss how they might change their plans to address their diabetes concern this week.

For more tips: Find *Planning to Make Healthy Changes* in the CO-IMPACT binder or website.

Update on Checking Sugars: Your partner reported that they checked their sugar 5 days in the last week.

Update on Checking Blood Pressure: Your partner reported that they checked their blood pressure 3 days in the last week.

Issues that were Identified

During the call, we identified issues that may interfere with good diabetes management or increase the risk of diabetes complications. For each issue that was identified, we asked your partner how important it is for them to make a plan to address this issue.

Issues your Partner Identified as Important

Your partner identified the following issue(s) as ones that are important for them to address this week. They were prompted to make an action plan for this problem. Ask if your partner would like to discuss their action plan to address the issue(s), and ask how they would like you to help.

ISSUE: Your partner reported that he had a systolic blood pressure less than 100 at least once in the last 2 weeks.

WHAT IT MEANS: If blood pressure goes too low, your partner may get dizzy or fall down.

HOW YOU CAN HELP:

- ♦ Help your partner figure out led to a low blood pressure. They can start by writing on their log the low pressure reading and time of day, how they felt, and how they ate and took their medication that day.
- ♦ If they are having low blood pressures more than once or twice, recommend that they call their primary care team to see if they need to adjust their medications or be evaluated.

Issues your Partner Identified as Not Important

ISSUE: Your partner reported that he needs a refill of one or more of his medicines or diabetes supplies.

WHAT IT MEANS: Keeping medicines filled and available can help avoid gaps in taking daily medications that help ensure that people with diabetes stay as healthy as possible. Your partner needs to refill his prescriptions regularly so that he never misses a dose.

HOW YOU CAN HELP:

- Ask your partner if you can help them get their medication or supplies refilled.

- Suggest that your partner write a note on the calendar to refill their medication 2 weeks before it will run out. It may also help to request a 90 day supply instead of a 30 day supply of routine medications or supplies.
- Remind your partner that there are several ways to get a refill - including by mail, phone, or website.

For more tips: Go to the Medications section on the CO-IMPACT website at http://hood.intervisionmedia.com/rosland/#resources?page=medications or in your Patient Partner's CO-IMPACT handbook.
