# Supplementary material for: Engaging family supporters of adult patients with diabetes to improve clinical and patient-centered outcomes: study protocol for a randomized controlled trial
Source: Trials. 2018 Jul 24;19:394. doi: 10.1186/s13063-018-2785-2 (PMC6057090; doi:10.1186/s13063-018-2785-2)
Supplement: Supplementary file 5 — Visit planning worksheet. (PDF 209 kb) [file 13063_2018_2785_MOESM5_ESM.pdf]

Visit Planning Worksheet for Primary Care Appointments

Next Appointment

Date:	Time:
Provider name:	
Labs/tests pre-ordered by primary care provider:	

Health Events since the last primary care appointment*

*Examples: visits to non-VA doctors or hospitals, illnesses, changes patient to health regimen
If you completed the Patient Event Log [hyperlinked], bring it with you to the appointment.

My questions for my primary care team:

Some Common Topics	
▪ Symptoms<br>▪ Using medicines<br>▪ Medication side effects<br>▪ High sugars<br>▪ Low sugars<br>▪ Controlling blood pressure<br>▪ Quitting smoking<br>▪ Healthy eating<br>▪ Physical activity<br>▪ What might happen in the future<br>▪ Getting things done at the VA<br>▪ Referrals or consults	

Information I will bring from home:

- ☐ Blood sugar log
- ☐ Blood pressure log
- ☐ Medication list or log
- ☐ Notes or results from non-VA medical visits
- ☐ Other:

Role I would like my Care Partner to play in my visit (if Care Partner is coming to appointment with me):

____

Available for Use with Permission from the Authors
