# Supplementary material for: Engaging family supporters of adult patients with diabetes to improve clinical and patient-centered outcomes: study protocol for a randomized controlled trial
Source: Trials. 2018 Jul 24;19:394. doi: 10.1186/s13063-018-2785-2 (PMC6057090; doi:10.1186/s13063-018-2785-2)
Supplement: Supplementary file 6 — Data management and security, safety monitoring, and adverse events. (DOCX 18 kb) [file 13063_2018_2785_MOESM6_ESM.docx]

Data Management:

Most survey responses will be entered directly into customized database forms by research assistants while conducting interviews. For any surveys completed on paper, responses are double entered into the database by two different research assistants, and any discrepancies are examined and corrected. The database limits data entry to acceptable value ranges. A Data Monitoring Committee is not required for this low-risk, single site study.

Data Security:

Study data will be stored on a protected and restricted sever. Data captured on paper will be stored in secure, locked cabinets in the research office space. Assessment data will be linked to the participant’s study ID number but separated from personally identifiable information. Visit summaries accessible to intervention-assigned participants will be hosted on a server that is compliant with the Federal Risk and Authorization Management Program. All patient data will be de-identified. Patients in the research project will sign an informed consent document which details how their data will be transmitted and stored. Access to the portal is password protected, using unique ‘strong’ passwords.

Safety Monitoring and Adverse Events:

The study will adhere to a pre-specified Data and Safety Monitoring Plan reviewed by the study IRB. All AEs, problems, and protocol deviations/violations will be brought to the immediate attention of the Principal Investigator, who will be responsible for reporting all serious adverse events (SAEs), adverse events (AEs), and serious problems, in a manner and time-course defined by the study site’s IRB policy.
